# Evaluating the implementation of person-centred care and simulation-based learning in a midwifery education programme in the Democratic Republic of Congo: a study protocol

**DOI:** 10.1080/16549716.2024.2370097

**Published:** 2024-06-25

**Authors:** Frida Temple, Ewa Carlsson Lalloo, Marie Berg, Urban Berg, Alumeti Munyali Désiré, Olivier Nyakio, Aline Mulunda, Malin Bogren

**Affiliations:** a Institute of Health and Care Sciences, Sahlgrenska Academy, University of Gothenburg, Gothenburg, Sweden; b Centre for Person‐Centred Care (GPCC), University of Gothenburg, Gothenburg, Sweden; c Faculty of Caring Science, Work Life and Social Welfare, University of Borås, Borås, Sweden; d Faculty of Medicine and Community Health, Evangelical University in Africa, Bukavu, Democratic Republic of Congo; e Institute of Clinical Sciences, Sahlgrenska Academy, University of Gothenburg, Gothenburg, Sweden; f UNFPA DRC, United Nations of Population Fund, Kinshasa, Democratic Republic of Congo

**Keywords:** Midwifery education, person-centred care, simulation-based learning, childbirth care, Central Africa, implementation science

## Abstract

**Background:**

Investing in midwives educated according to international standards is crucial for achieving Sustainable Development Goals in maternal and newborn health. Applying a person-centred care approach and using simulation-based learning to improve the learning experience for midwifery students may enhance the quality of childbirth care. This protocol describes a study evaluating the implementation of person-centred approach and simulation-based learning in childbirth as part of a midwifery education programme at the Evangelical University in Africa, DRC.

**Methods:**

The research will be exploratory and guided by an implementation research framework. Ethical approval has been obtained. Facilitators working at the programme’s five clinical practice sites will be trained in: 1) Introducing person-centred childbirth care using a training programme called‘Mutual Meetings’; and 2) integrating simulation-based learning, specifically by using the three courses: Essential Care of Labor, Bleeding after Birth, and Vacuum Extraction. Data will include interviews with midwifery students, facilitators and clinical preceptors, and maternal and neonatal outcomes from birth registers.

**Discussion:**

By integrating a validated and culturally adapted person-centred care training programme and simulation-based learning into a midwifery education programme and clinical practice sites, the findings from the study anticipate an improvement in the quality of childbirth care. Training facilitators in these methodologies aim to effectively mitigate maternal and neonatal adverse outcomes. The findings are expected to provide valuable recommendations for governments, policymakers, and healthcare providers in the DRC and beyond, contributing to significant improvements in midwifery education and aligning with global health priorities, including the Sustainable Development Goals.

**Trial registration:**

The study was registered retrospectively with the ISRCTN registry on the 23rd of February 2024. The registration number is: ISRCTN10049855.

## Paper context

- **Main findings:** It is anticipated that the implementation of both person-centred care and simulation-based learning in a midwifery education programme will improve the quality of care in childbirth practice.
- **Added knowledge:** The use of facilitators has the potential to enhance the implementation of person-centred care and simulation-based learning in a midwifery education programme, both at campus and in clinic.
- **Global health impact for policy and action:** The expected findings could inform global health policy development and practice, promising advancements in midwifery education and consequently enhance the maternal and newborn health outcomes.

## Background

### The impact of professional midwives on quality maternal and new-born healthcare

Barriers to achieving good quality midwifery education remain, particularly in low-income countries including the Central Africa Region. Inadequately educated staff may negatively influence the safety of mothers and newborns during labor, birth, and the early postpartum period [1]. This emphasizes the urgency of addressing the global shortfall of midwives educated according to international standards, and which is crucial for achieving the Sustainable Development Goals [2–4]. These goals aim at improving maternal and newborn health, consequently contributing significantly to the reduction of maternal and neonatal mortality rates, as well as stillbirths [5].

### Challenges in maternal and new-born health care in the Democratic Republic of Congo

The Democratic Republic of Congo (DRC), in Central Africa, confronts challenges in maternal and newborn health, with a maternal mortality rate of 547 per 100 000 live births and newborn mortality rate of 28 per 1000 live births. These rates are among the highest in the world, despite 80% of births taking place in healthcare facilities [6,7]. Complications arising from pregnancy and childbirth are the leading causes of morbidity and mortality among women of reproductive age [7]. Escalating cesarean section rates is a concern in the DRC, as well as globally, and addressing it confronts its own set of challenges [8]. A woman’s interaction with maternity care providers significantly influences the quality of care and thus is crucial for maternal and new-born health. In resource-constrained settings, including the DRC, healthcare providers face system deficiencies that may lead to mistreatment of women and violation of human rights [9].

### Person-centred childbirth care

Person-centred care aims to enhance conditions for equitable healthcare by fostering mutual respect and collaborative health creation, transforming patients from passive recipients into active partners [10,11]. Person-centred care is based on an ethical framework where the starting point is the dialogue between patient and healthcare providers which lays the foundation for a partnership – a mutual and respectful relationship between the patient and the healthcare providers [12].

The integration of a person-centered approach into maternal and newborn healthcare has the potential to reduce maternal and neonatal morbidity and mortality, as there is evidence that once person-centred maternity care is implemented, there are less maternal and neonatal complications [13]. Thus, implementing person-centered maternity care has proven to positively impact maternal physical and mental health, newborn well-being, and experiences and increasing equity of care [14,15].

Successful implementation of person-centred care requires contextual adaptation of the innovation to the specific environment in which it is being introduced [16]. The implementation is not a straightforward task; it necessitates a shift in organizational culture, encompassing healthcare providers’ knowledge, attitudes, and behaviors [17]. The Integrated Promoting Action on Research Implementation in Health Services (i-PARIHS) framework, crafted to navigate the complexities of research implementation, underscores the vital role of a facilitator [18]. Positioned as the ‘core ingredient,’ facilitation, encompassing both a specific role and a set of interactive, context-responsive actions, is pivotal in enabling successful implementation and addressing emerging barriers within the implementation context [19]. Earlier experiences of implementing person-centred care in other health care contexts has shown to be fragmented and highly delivered by individuals. More research is therefore needed to understand how to effectively implement in a higher education institution [20].

### Simulation-based learning as a tool to improve clinical competence

To improve clinical competence, simulation-based learning can be used; a form of active pedagogy whereby simulated patient care scenarios helps learners acquire knowledge and skills that increase patient safety [21,22]. The evidence shows the knowledge enhancement experienced by students exposed to simulation-based learning, firmly establishing it as a cornerstone in refining healthcare providers’ practices [23]. The impact of student-centred simulation-based learning extends beyond the classroom, cultivating professional development and supporting learners in their transition from students to healthcare professionals [24]. For midwifery students, this approach serves as more than just an educational tool; it becomes a transformative experience, fostering problem-solving and communication skills within an environment mirroring real-life situations, ultimately enhancing learning in a safe environment [23,25]. In addition, simulation-based learning is cost-effective when implemented in clinical settings [26] making it particularly beneficial for low-income settings. Research indicates that simulation-based team training among healthcare providers leads to sustained enhancements in responding to critically deteriorating in-patients, resulting in improved patient outcomes [26]. The integration of regular on-site simulation training for healthcare providers, such as midwives, involving staff in routine clinical care, is beneficial across various acute specialities including maternal health care.

### The existing knowledge on midwifery education in Democratic Republic of Congo

The DRC exposes a shortage of midwives meeting international standards. Previous research argues that midwifery curricula according to international standards are deficient in key competencies necessary for delivering high-quality childbirth care in DRC [27]. Another study from DRC identified a shortage of simulation-based learning activities and inadequate materials at clinical sites for midwifery education programmes [28]. Notably, both midwifery educators and clinical preceptors acknowledged insufficient competencies, affecting their ability and quality of instruction [29]. Drawing from these findings, and in response to the identified challenges, a midwifery education programme is being implemented at the Evangelical University in Africa (a conversion programme from A1 nurses and midwives), situated in eastern DRC. To overcome the challenges observed in the existing education system the programme adheres to the national curriculum with the specific profiles on person-centred care and simulation-based learning [27].

A contextual study investigated the factors influencing the implementation of such midwifery education programme in the DRC, considering the diploma-level status of existing midwifery education. The findings underscored the importance of higher education for midwives. Moreover, the study revealed that adopting person-centred care is considered innovative and necessary in the DRC, with the expectation that it would enhance the quality of maternal and newborn health care in the country. The results also emphasized that simulation-based training was considered to result in relevant competencies for midwives prior to clinical exposure and that educational materials are needed to ensure quality of the simulation-based training [30].

## Aim, hypothesis and design

### Aim

The overall aim of this research is to evaluate the implementation of a person-centred approach and simulation-based learning in childbirth care as part of the midwifery education program at Evangelical University in Africa in the Democratic Republic of Congo.

The specific aims are to

- validate and culturally adapt the Swedish person-centred care training programme ‘Mutual meetings’ in an under-resourced environment.
- to determine the effectiveness of training midwives and gynecologists to function as facilitators in the implementation of person-centred care and the use of childbirth simulation-based learning activities at the clinical practice sites for midwife students.

### Hypothesis

Our hypothesis is that the implementation of both person-centred care and simulation-based learning in a midwifery education programme by educators will increase the quality of care in childbirth practice, and thereby improve maternal and neonatal outcomes.

## Methods

### Research design

**Figure 1.**
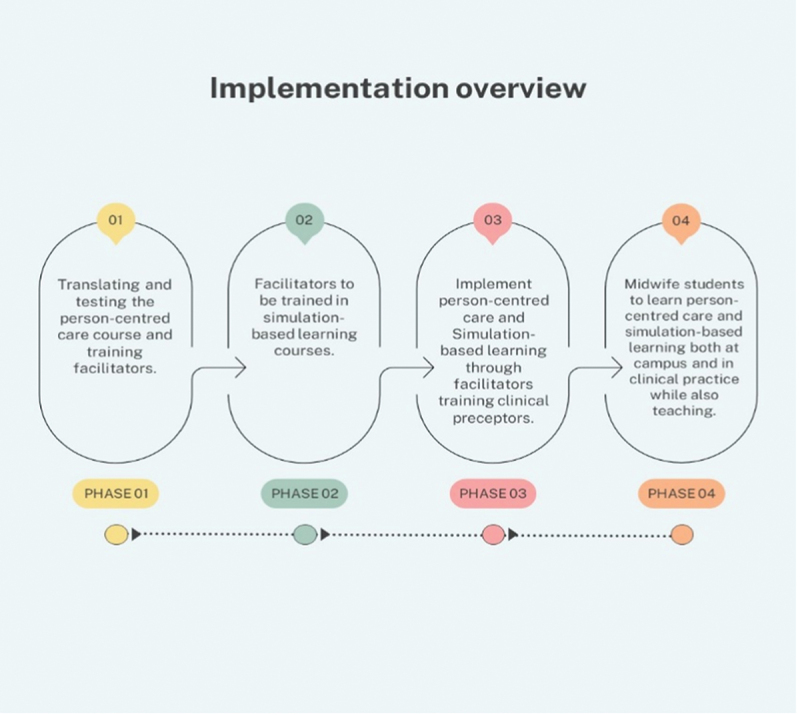
Implementation overview.

**Figure 2.**
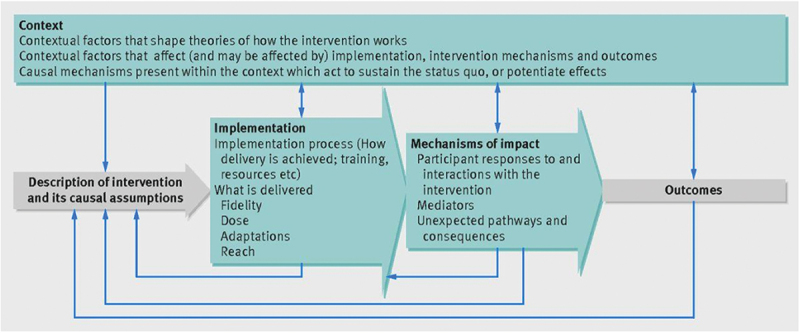
The evaluation framework by Moore et al. [31].

The evaluation of the implementation of both person-centred care and simulation-based learning in a midwifery education programme will initially be conducted separately. Subsequently, a combined evaluation will be carried out. The implementation of the two profiles was conducted in four phases (Figure 1). This evaluation process will utilize an exploratory design guided by the principles of an evaluation framework (Figure 2). This includes a description of the implementing process, and what is delivered (fidelity, dose, adaptions, and reach), the mechanisms of impact that influence the learning (responses, mediators, unexpected pathways, and consequences) [31], and the mother and child outcomes and adherence to the created implementation plans. Ethical approval has been attained by the National Ethical Committee in Sweden Dnr: 2023–07343–01 and the National Ethical Committee of Public Health in the South Kivu Province Register number: CNES 001/DPSK/219PP/2024 and CNES 035/DPSK/219PP/2022.

### Setting

The Evangelical University in Africa is situated in Bukavu in eastern DRC. In October 2022, a 90 credits midwife education programme started at the faculty of Medicine and Community Health for nurses, having a 3-year education on university level. The programme is at the bachelor level and adheres to the national curriculum. However, it is further innovatively designed with a profile of person-centred care and the integrated use of simulation-based learning pedagogy and materials. The clinical practice sites are situated at five hospitals located in the South and North Kivu provinces in eastern DRC.

### Intervention

The intervention involves integrating two profiles into a midwifery education programme aligned with the national curriculum, namely person-centred care, and simulation-based learning. The strategy used to implement and integrate the two profiles into clinical practice is training facilitators from each clinical site to further ensure effective and sustained implementation.

### Profile person-centred care

**Figure 3.**
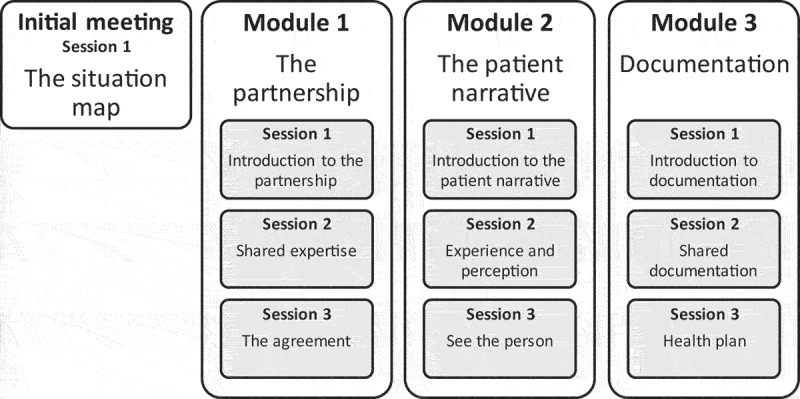
Overview of the person-centred care training programme - mutual meetings.

Based on scientific evidence, an ethical framework, and clinical practice, the University of Gothenburg Centre for Person-Centred Care (GPCC) has developed a model for person-centred care, constituting three routines: i) Initiating a partnership, ii) Establishing an agreement between patient and healthcare provider, and iii) Safeguarding the partnership through documentation [32,33], Figure 3. To support health care units implementing the person-centred care model, GPCC has developed an online training programme, ‘MedMänniska’ [Mutual Meetings] that supports professionals´ learning and training and equipping them with tools to implement person-centred care in their respective health care context [34]. This training programme will be implemented in the midwifery education programme and consists of ten lessons focusing the three routines in person-centred care. Its didactive method includes theory, individual reflections, common discussions, and exercises. Mutual Meetings, to our knowledge, have not been reported to be used in any low-income settings.

The implementation of person-centred care using the strategy of training facilitators to support the implementation includes the following steps:

- Translate the Swedish version of ‘MedMänniska’ into French.
- The French version of ‘Mutual Meetings’ will next be translated into French and independently be reviewed and culturally adapted by two native Congolese with knowledge and experience in healthcare in the DRC.
- ‘Mutual Meetings-French’ will be tested by its feasibility at a three-day workshop with 30 participants including educators at the university, leaders and staff at the clinical practice sites, provincial healthcare leaders and representatives for the social community. They will also attend a follow-up workshop where a training of the final version of the Translated cultural adapted Mutual Meetings will lead to the participants becoming facilitators.
- After validation, person – centered care will be implemented in the five targeted clinical sites through ‘Mutual Meetings-French’ facilitators.

### Profile simulation-based learning

Theoretical knowledge, simulation-based learning, and practical clinical training will be integrated in the midwifery education programme at both campus and clinical practice sites. As part of this simulation-based education at clinical practice, selected gynecologists and midwives will be trained to become facilitators. This study concerns implementation of three selected simulation-based learning courses: i) Essential Care for Labor and Birth (ECLB), ii) Vacuum Assisted Birth (VAB) and iii) Bleeding After Birth Complete (BABC).

The implementation strategies include the following steps:

- Facilitators (*n* = 12) from each clinical site (*n* = 5) will be trained in the courses ELCB, VAB, and BABC during a 4-day facilitator training and will after completion of the courses train their colleagues at their respective maternity units (*n* = 50). The training will use the low-dose, high-frequency approach. In total, about 50 healthcare providers will be capacitated to function as preceptors for midwife students, using the ELCB, VAB, and BABC courses.
- The maternity care units (*n* = 5) will each receive necessary pedagogic tools related to the ECLB-, VAB-, and BABC courses including posters, flipcharts, and facilitator guides for each course as well as birth simulator mannequins and vacuum extractors.
- The clinical preceptors will subsequently train the midwifery students, using the low-dose, high-frequency approach aiming for weekly trainings in clinical setting through simulation-based training by utilizing the education material and mannequins provided in the ELCB, VAB and BABC courses. The person-centred care approach will be included in the simulation-based learning scenarios.

### Monitoring visits

A local project coordinator will do weekly follow-ups and visits with each of the five clinical practice sites to ensure effective implementation of the individual implementation plans. The objective of the monitoring visits is to support and enhance the implementation of person-centred care and simulation-based learning at the clinical sites by ensuring management approval, providing continuous support to facilitators, and maintaining effective communication throughout the project. The local project coordinator will work as an external facilitator while the trained facilitators based at each clinical practice site will work as internal facilitators. Internal facilitators are identified as facilitators working in the implementation site [18] and the external facilitator refers to facilitation that comes from a change agency outside of the implementation site [35].

### Data collection

Both qualitative and quantitative data will be collected through a multi-faceted approach encompassing focus group interviews, key informant interviews, and register data. Focus group interviews will be used to retrieve a full picture and diversity of the participants agreeing and disagreeing with different sentiments. A few individual interviews will be conducted when there is a need for more in-depth information. Study participants will include trainers for person-centred care (*n* = 2), trained facilitators in person-centred care (*n* = 30), trained facilitators in simulation-based learning (*n* = 12), clinical preceptors (*n* = 50), and midwifery students (*n* = 30).

To capture feasibility, clarity, usability, and relevance [31] of the intervention’s profile on person-centred care, audio-recorded focus group interviews will be conducted with the workshop participants, using an interview guide with semi-structured questions. The questions concern how the training programme was perceived, the pedagogics and what the participants have gained from the course to become facilitators after the training programme. They also address the relevance of the training programme and questions related to the implementation of person-centred care.

For the intervention´s profile of simulation-based learning, focus group interviews will be held using an interview guide covering the following components of the process evaluation framework: fidelity, dose/exposure, reach, acceptability [31]. The questions concern how the course was perceived, the pedagogics, and if the participants feel ready to become facilitators after the course. For the clinical preceptors, the questions concern how the preceptors were trained by the facilitators, how the simulation material has been used in the mentoring of the midwife students, and how this has been useful when caring for patients and in supervising the midwifery students.

For the evaluation of the two profiles combined, focus group interviews will be held with the students of the first midwife program (*n* = 30), the interview guide will cover the following components of the process evaluation framework mechanisms of impact: Participants responses and interactions with the interventions, mediators, unexpected pathways and consequences [31].

To further investigate the effect of the intervention’s impact on the quality of care, particularly the influence on maternal and neonatal outcomes at the five clinical practice sites, we will collect quantitative data from the birth register, Table 1. All births for the years 2022 and 2024 will be included.

**Table 1.** Variables collected in birthregisters.

Mode of birth: Jan–Dec 2022–Jan–Dec 2024
Vaginal spontaneous birth
Caesarian birth emergency
Planned caesarian birth
Maternal mortality
Neonatal mortality
Obstetrical complications
Still births Fresh
Postpartum Hemorrhage >500 ml
Vacuum assisted birth

### Data analysis

Analysis of qualitative data from focus group interviews will be based on transcribed interviews, applying deductive qualitative content analysis principles [36]. The process evaluation framework will guide the analysis, emphasizing key components such as Implementation, Mechanisms of Impact, and Outcomes [31].

The maternal and neonatal outcomes will be compared between the two time periods, where Fisher’s exact test will be used for dichotomous variables and the Chi-square test for non-ordered categorical variables. The mean differences with 95% confidence intervals and p-values will be the main results in the comparative analysis. All the tests will be two-sided and conducted at the 5% significance level.

## Discussion

### Significance and scientific novelty of the research

It is expected that this research will fill knowledge gaps as discussed below:

- The introduction of person-centred care within a midwifery education programme and its clinical practice sites in an African low-income setting represents a unique opportunity to pioneer and gain practical experience in implementing person-centred care in challenging healthcare environments. It is anticipated that the validated programme on person-centred care can support the implementation of person-centred childbirth care within the DRC maternal and newborn health care context. By disseminating this programme, a culture of person-centred care can be fostered both in DRC and beyond.
- The unexplored combination of training person-centred care included in simulation-based learning activities, underscores the research team’s commitment to advancing knowledge and improving healthcare practices [27,29]. We anticipate that this study will provide additional evidence to guide effective implementation combining person-centred care and simulation-based learning into a midwifery education programme that contributes to high-quality care during childbirth.
- Current global trends and projections anticipate that almost 30% of women worldwide will undergo caesarean section births by 2030 [37]. Simulation-based learning, specifically the course on vacuum extraction, can support the midwives to increase their competence in advocating for vaginal births, with an emphasis on spontaneous, physiological births, as well as integrating safe instrumental methods on their practice [38].
- This research employs facilitators as a strategy, integrating external and internal facilitators, to implement interventions at clinical sites; a strategy proven effective in past studies [18,35]. It can be argued that such an approach can be useful in post-colonial countries, as it emphasizes local ownership and contextual relevance of the interventions, rather than relying solely on external influence.
- Using an implementation science framework [31] in midwifery education is a new approach that enables exploring how person-centered care and simulation-based learning are implemented and their mechanisms of impact and outcomes. These findings can provide lessons learned and guide other education institutions that want to implement a similar programme.

## Impact of the research

- At policy level: Through the results of this research, we hope for successful implementation of person-centred care in midwifery education and clinical practice in low-income settings. This could pave the way for policy recommendations promoting its widespread adoption across healthcare professions in the DRC and other similar contexts.
- At education level: We anticipate that the validated training programme ‘Mutual Meetings’ serves as a foundation and could be extended beyond midwifery to enhance healthcare education [20]. The new knowledge generated provides an opportunity for governments, researchers, and policymakers in the DRC and elsewhere to integrate person-centred care and simulation-based learning into midwifery education programmes, contributing to improved quality of care.
- In quality of care: If the intervention has positive effects on maternal and newborn health outcomes, such as increased proportion of vaginal birth and reduced morbidity and mortality and thus improving women’s childbirth experiences, we can through this intervention prove that equipping midwives with competence in person-centred care and simulation-based learning offers a strategic approach to improving maternal and newborn health.

## Authors’ contributions

All authors (FT, ECL, MB, UB, AMD, ON, AM and MBO) participated in the development and planning of the research. FT coordinated the writing of the protocol. The work was supervised by MBO, ECL, MB and UB. All authors provided feedback and approved the final manuscript.

## Disclosure statement

No potential conflict of interest was reported by the author(s).

## Ethics approval and consent to participate

Ethical clearances were obtained from: the National Ethical Committee in Sweden Dnr: 2023–07343–01 and the National Ethical Committee of Public Health in the South Kivu Province Register number: CNES 001/DPSK/219PP/2024 and CNES 035/DPSK/219PP/2022. All methods, including procedures to obtain informed consent from all study participants, will be carried out according to relevant regulations as well as the Helsinki Declaration, 1975. The consent for publication is clearly stated in the consent form and will be obtained by the participants in the studies.
